# Aneurysm-Specific miR-221 and miR-146a Participates in Human Thoracic and Abdominal Aortic Aneurysms

**DOI:** 10.3390/ijms18040875

**Published:** 2017-04-20

**Authors:** Premakumari Venkatesh, Julie Phillippi, Sasanka Chukkapalli, Mercedes Rivera-Kweh, Irina Velsko, Thomas Gleason, Paul VanRyzin, Seyed Hossein Aalaei-Andabili, Ravi Kiran Ghanta, Thomas Beaver, Edward Kar Leung Chan, Lakshmyya Kesavalu

**Affiliations:** 1 Department of Periodontology, University of Florida, Gainesville, FL 32610, USA; vapg_1@yahoo.com (P.V.); SChukkapalli@dental.ufl.edu (S.C.); mrivera4@ufl.edu (M.R.-K.); irinavelsko@gmail.com (I.V.); 2 Department of Cardiothoracic Surgery, University of Pittsburgh School of Medicine, Pittsburgh, PA 15219, USA; phillippija@upmc.edu (J.P.); gleasontg@upmc.edu (T.G.); paul@vanryzin.org (P.V.); 3 Departments of Surgery, University of Florida, Gainesville, FL 32610, USA; hossein.aalaei@surgery.ufl.edu (S.H.A.-A.); thomas.beaver@surgery.ufl.edu (T.B.); 4 Michael E. Debakey Department of Surgery, Baylor College of Medicine, Houston, TX 77030, USA; Ravi.Ghanta@bcm.edu; 5 Department of Oral Biology, University of Florida, Gainesville, FL 32610, USA; ECHAN@dental.ufl.edu

**Keywords:** thoracic aortic aneurysm, abdominal aortic aneurysm, microRNA

## Abstract

Altered microRNA expression is implicated in cardiovascular diseases. Our objective was to determine microRNA signatures in thoracic aortic aneurysms (TAAs) and abdominal aortic aneurysms (AAAs) compared with control non-aneurysmal aortic specimens. We evaluated the expression of fifteen selected microRNA in human TAA and AAA operative specimens compared to controls. We observed significant upregulation of miR-221 and downregulation of miR-1 and -133 in TAA specimens. In contrast, upregulation of miR-146a and downregulation of miR-145 and -331-3p were found only for AAA specimens. Upregulation of miR-126 and -486-5p and downregulation of miR-30c-2*, -155, and -204 were observed in specimens of TAAs and AAAs. The data reveal microRNA expression signatures unique to aneurysm location and common to both thoracic and abdominal pathologies. Thus, changes in miR-1, -29a, -133a, and -221 are involved in TAAs and miR-145, -146, and -331-3p impact AAAs. This work validates prior studies on microRNA expression in aneurysmal diseases.

## 1. Introduction

Early detection and risk stratification of aortic aneurysms is essential to reduce risk of catastrophic rupture and dissection, which carries high risk of morbidity and mortality [1]. Aortic aneurysms occur secondary to pathologic remodeling of the aortic wall and are frequently associated with hypertension [2], atherosclerosis [3,4] in the abdominal aorta, and bicuspid aortopathy [5], or connective tissue disorders [6] in the ascending thoracic aorta. The pathogenesis of aneurysms differs based on the involved aortic segment. Ascending thoracic aortic aneurysms (TAAs) frequently result from a non-inflammatory process of cystic medial degeneration including smooth muscle cell (SMC) loss and elastin fragmentation [7]. Conversely, descending TAAs and abdominal aortic aneurysms (AAAs) involve elastic degeneration, but this can occur via an inflammatory and atherosclerotic process. TAAs and AAAs each exhibit degeneration of the extracellular matrix (ECM) involving increased expression of matrix metalloproteinases (MMPs) and/or decreased expression of MMP antagonists, the tissue inhibitors of MMPs (TIMPs). Identification of biomarkers that are driven by aortic remodeling is important to allow for early identification and risk stratification. In addition, biomarkers may represent therapeutic targets to prevent aneurysm expansion.

In recent years, microRNAs (miRNAs) have emerged as critical regulators of many key cellular functions and have been linked to a variety of pathologies, including cardiovascular diseases [8]. miRNAs play a vital role in the development of ventricular myocardium, cardiac outflow tract alignment, chamber septation, and in the maintenance of healthy heart function [9,10,11]. Some studies have identified miRNAs expression profiles that are linked to SMC functions such as differentiation [12] or proliferation [13], whereas other miRNAs are expressed in endothelial cells, and alterations have been shown to impact angiogenesis and vascular remodeling [14]. Also, important for improving our understanding of cardiovascular pathophysiology is the mounting evidence of specific functions of miRNAs, such as miR-29, which participates in pathological ECM remodeling [15,16,17]. This growing understanding of miRNA control of ECM perturbations and role in vascular remodeling suggest that miRNAs have direct contributions to aneurysm pathophysiology.

Prior studies have reported alterations in the expression of several miRNAs in the setting of aortic diseases [16,18,19,20]. No study has yet directly compared miRNA expression profiles between AAAs and TAAs versus non-aneurysmal specimens simultaneously. We hypothesized that aneurysm pathologies have a unique miRNA signature depending on their anatomic location. Therefore, we comprehensively analyzed the expression of fifteen miRNAs in human TAA, AAA, and non-aneurysmal specimens using quantitative reverse transcription polymerase chain reaction (qRT-PCR). This work revealed unique expression patterns of miRNAs in TAAs and AAAs with some overlapping alterations in both aortic pathologies. This work adds to a growing body of knowledge and highlights the need for understanding the function of these important regulators of vascular biology in the setting of disease.

## 2. Results

### 2.1. Patient Demographics

We did not observe any significant age differences between TAA and AAA patient samples, but non-aneurysmal controls were collected from younger patients (control, 57 ± 4 years; TAA, 65 ± 5 years; AAA, 66 ± 1 years) (Table 1).

### 2.2. Analysis of Candidate miRNA Expression

**Figure 1:**
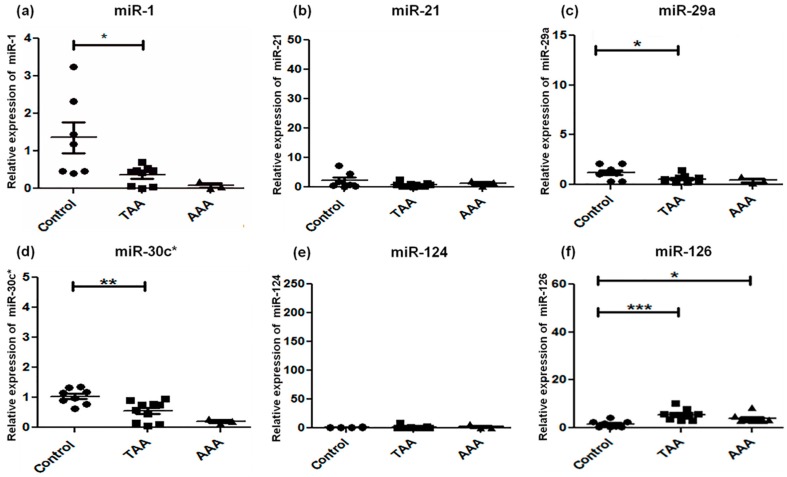
Alterations in microRNA profiles of miR-1 (**a**), miR-21 (**b**), miR-29a (**c**), miR-30c-2* (**d**), miR-124 (**e**), and mir-126 (**f**) by qRT-PCR. Expression levels in TAA (*n* = 11) and AAA (*n* = 3) as compared with non-aneurysmal control aorta (*n* = 8). The relative amount of each miRNA was normalized to small nucleolar RNA (RNU44). Statistically significant differences: * *p* < 0.05, ** *p* < 0.01, *** *p* < 0.001.

**Figure 2:**
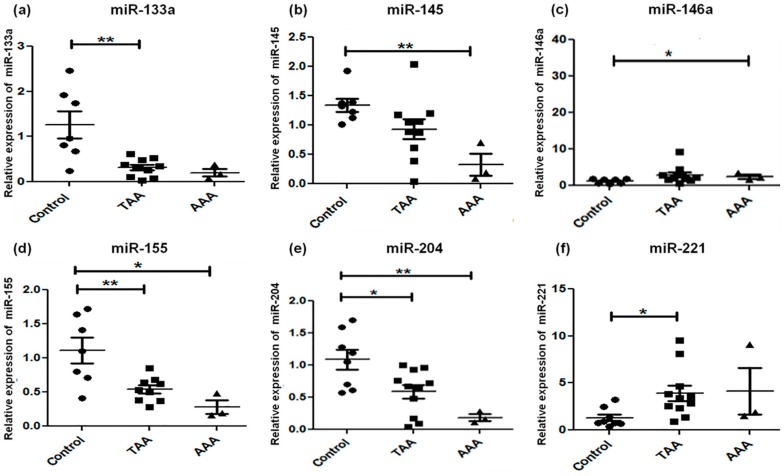
Alteration in microRNA profiles of miR-133a (**a**), miR-145 (**b**), miR-146a (**c**), miR-155 (**d**), miR-204 (**e**), and miR-221 (**f**) by qRT-PCR. Expression levels in TAA (*n* = 11) and AAA (*n* = 3) as compared with non-aneurysmal control aorta (*n* = 8). The relative amount of each miRNA was normalized to RNU44. Statistically significant differences: * *p* < 0.05, ** *p* < 0.01.

**Figure 3:**
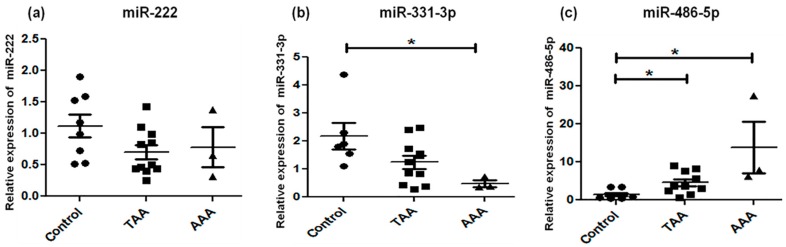
Alteration in microRNA profiles of miR-222 (**a**), miR-331-3p (**b**), and miR-486-5p (**c**) by qRT-PCR. Expression levels in TAA (*n* = 11) and AAA (*n* = 3) as compared with non-aneurysmal control aorta (*n* = 8). The relative amount of each miRNA was normalized to RNU44. Statistically significant differences: * *p* < 0.05.

Expression of the fifteen miRNAs was analyzed by qRT-PCR in TAA, AAA, and control (non-aneurysmal) aortic specimens (Table 2; Figure 1, Figure 2 and Figure 3). The selection of these 15 candidate miRNAs was based on their reported fold changes associated with TAA and AAA vs. controls reported in the literature. miR-1, miR-133a, and miR-30c-2* were found to be downregulated in TAA when compared with control specimens (*p* < 0.05), whereas for downregulation of miR-30c-2* in AAA compared to control there were no changes in the expression of miR-1, -133a, and -29a in AAA (Figure 1). miR-146a was upregulated and miR-145 significantly downregulated in AAA, whereas miR-21, -222, and -124 did not exhibit any alteration for either TAA or AAA (Figure 2). miR-221 was upregulated in TAA, while miR-204 was downregulated in TAA, whereas miR-145 and miR-204 were downregulated in AAA (*p* < 0.01) (Figure 2). The expression pattern of miR-331-3p was downregulated in AAA, while miR-486-5p showed upregulation in both TAA and AAA when compared to control specimens (Figure 3). Some miRNAs showed overlapping expression profiles in both TAA and AAA (Table 2); miR-126 and -486-5p were upregulated in TAA and AAA, whereas miR-30c-2*, -155, and -204 were all downregulated in TAA and AAA when compared with control specimens (Table 2). miR-221 expression level specifically upregulated in TAA and did not change in the AAA group.

### 2.3. Microarray Expression Analysis

**Figure 4:**
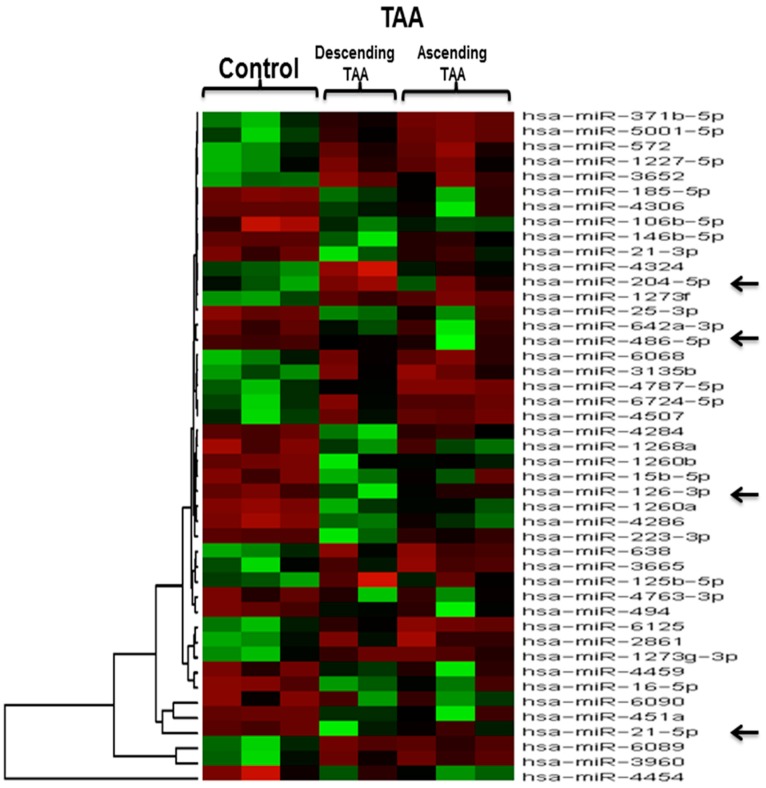
Heat map (Human miRNA microarray) depicting 45 differentially expressed miRNAs in TAA. Differentially expressed miRNAs (*p* < 0.05 and absolute value (fold change) > 1.5) in TAA specimens as compared with non-aneurysmal control aorta is shown. Arrows indicate the miRNAs whose expression was similar in qRT-PCR analysis. TAA (*n* = 5) and normal (*n* = 3).

**Figure 5:**
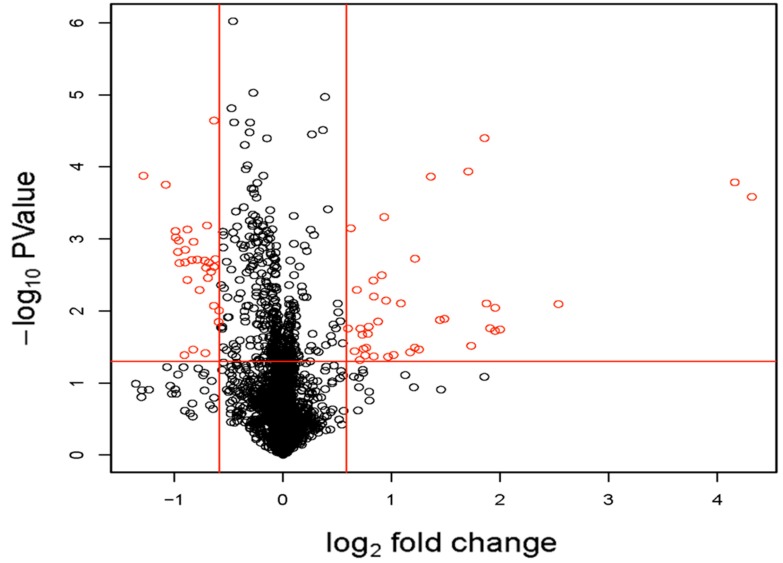
Volcano map demonstrating differences in miRNA expression levels in TAA by microarray. The log-fold change is plotted against the log odds of differential expression. The miRNAs with significant differences in expression levels between the TAA (*n* = 5) and control (*n* = 3) groups (*p* < 0.05) after Benjamini-Hochberg correction are indicated.

Microarray analysis of three control aortic specimens and five TAA tissue samples revealed 45 differentially expressed miRNAs between the control aorta and the TAA at *p* < 0.05 and fold change >1.5. These differentially-expressed miRNAs consisted of 24 miRNAs with increased expression in the TAA (miR-15b-5p, miR-16-5p, miR-21-3p, miR-21-5p, miR-25-3p, miR-106b-5p, miR-126-3p, miR-146b-5p, miR-185-5p, miR-223-3p, miR-451a, miR-486-5p, miR-494, miR-642a-3p, miR-1260b, miR-1260a, miR-1268a, miR-4284, miR-4286, miR-4306, miR-4454, miR-4459, miR-4763-3p, miR-6090) and 21 miRNAs with decreased expression in the TAA (miR-125b-5p, miR-204-5p, miR-371b-5p, miR-572, miR-638, miR-1227-5p, miR-1273g-3p, miR-1273f, miR-2861, miR-3135b, miR-3652, miR-3665, miR-3960, miR-4324, miR-4507, miR-4787-5p, miR-5001-5p, miR-6068, miR-6089, miR-6125, miR-6724-5p) when compared with non-aneurysmal control specimens, as described in the heat map in Figure 4 and in the volcano plot in Figure 5. The differentially regulated miRNAs in microarray analysis with their fold change and *p* values are shown in Table 3 (upregulated) and Table 4 (downregulated). Further, we compared five TAA samples that were evaluated by both qRT-PCR and microarray analysis to examine if any correlations exist among two molecular methods of expression analysis. Interestingly, miR-126 and miR-486-5p were found to be upregulated commonly by both qRT-PCR and microarray in TAA patients, while miR-204 was found to be downregulated, indicating the validity of correlation among two different platforms of gene expression analysis.

## 3. Discussion

miRNAs are promising diagnostic and therapeutic targets for the management of cardiovascular diseases, but the current literature available on their role in TAA and AAA is still scarce. A few prior studies have identified altered miRNA expression patterns in aneurysmal aortic diseases, however, findings have been inconsistent. No prior study has compared expression between TAA and AAA, which are pathologically and clinically distinct diseases. Further prior studies done so far followed a conventional approach of screening by first microarray followed by validation using qRT-PCR. Our study used a non-conventional approach, as it examined the top 15 candidate miRNAs associated with TAA and AAA, and later further examined their expression pattern by stringent microarray analysis. In this study, we identified unique miRNA expression signatures for specific aneurysm locales as well as overlapping profiles in both pathologies, partially confirming prior work reported in the literature [15,16,18,19,21,22]. 

While TAA and AAA have differing miRNA expression profiles, there are select miRNAs with similar expression profiles in both aneurysm pathologies. These uniformities may help identify common mechanisms-of-action that give rise to both TAA and AAA. An upregulation of miR-126 was detected in TAA and AAA alike. Work by others revealed that miR-126 promoted angiogenesis by alleviating suppression of VEGF (vascular endothelial growth factor) and FGF-2 (fibroblast growth factor) signaling by Spred1 (Sprouty-related, EVH1 domain-containing protein 1) [23], while the influence of upregulated miR-126 in aneurysm formation is not yet clear. Likewise, miR-155 has been linked to atherosclerotic lesions by limiting endothelial response to inflammatory cytokines [24]. The downregulation of miR-155 as a negative regulator of endothelial inflammatory response in AAA specimens is consistent with the inflammatory nature of AAA. Conversely, in the thoracic aorta, where medial degeneration occurs in the absence of inflammation, miR-155 deficiency may play an alternative role in aneurysm pathogenesis. In TAA and AAA, we also noted downregulation of miR-30c-2* which has been shown to participate in cardiac remodeling and fibrosis as a negative regulator of connective tissue growth factor (CTGF), a pro-fibrotic growth factor that promotes deposition of ECM components [25]. Hence, miR-30c-2* downregulation in TAA and AAA likely impacts composition of the ECM. The upregulation of miR-486-5p noted in our TAA and AAA specimens suggest involvement of this miRNA in medial SMC contractile function as a factor influencing aneurysm formation. The similarities of certain miRNA expression profiles in both TAA and AAA suggests a role for them in the common pathways of aneurysm progression such as degeneration of the collagen and elastin matrix and defective SMC function in the aortic wall. 

In TAA specimens, we noted an upregulation of miR-221 that was not seen in AAA. This outcome was somewhat surprising, given that miR-221 regulates the proliferation of SMCs during neointimal hyperplasia [21], which has not been well described for TAAs. In opposition, miR-1, -29a, and -133a were found to be downregulated in TAA patients only. Jones and colleagues identified a similar downregulation of these miRNAs in 30 human TAA samples [18]. Furthermore, they reported that downregulation of miRNA-29a in human TAA was dependent on aortic diameter and proteolytic activity [18]. In contrast, miR-29 upregulation was noted in animal models and human TAA specimens [15]. The miR-29 family is one of the most heavily studied miRNA families. Expression of miR-29a/b/c were all shown to be increased in fibullin-4-deficient mice [15], a model of TAA, and in fibrillin-1-deficient mice [22], a model of Marfan syndrome. It was further demonstrated that miR-29a directly influenced maintenance of ECM components including collagen and elastin [15]. Interestingly, the only miR-29 family member to exhibit increased expression in human TAA specimens examined was miR-29b [15]. Decreases in miR-133a expression level, as we confirmed here, likely play a role in matrix degradation and remodeling as MMP-2 and -9 were shown to be targets of miR-29a in vitro [18]. The study by Jones et al. also documented downregulation of miR-21, which we did not observe in our TAA specimens. 

In contrast to TAA, expression of miR-1, -29a, and -133a were unaltered in AAA specimens. An upregulation of miR-146 was noted in our AAA specimens. miR-146 has shown involvement in inflammatory diseases including AAA [16]. Downregulation of miR-145 and miR-331-3p were identified during analysis of AAA specimens. Downregulation of miR-145 via knock-down of *Dicer* in a mouse model led to decreased thickness of the aortic media, associated with reduced SMC differentiation [13]. The mechanism of action for miR-145 in AAA has yet to be elucidated, but may involve a negative influence on the contractile SMC phenotype [26,27,28,29] and potentially contribute to a dysfunctional matrix-cell unit and weaken vessel integrity. The exact functions of miR-331-3p in cardiac function and disease have not been established, and a role in aneurysm has not previously been studied. Since miR-331-3p downregulation has been associated with cancer [30], with the downregulation of miR-331-3p we noted in AAA specimens, we could speculate that miR-331-3p might play a role in cell cycle regulation of SMCs in the setting of AAA. While our study did not reveal an alteration in expression of miR-21 in AAA specimens, others have shown that over-expression of miR-21 in a mouse model of AAA abrogated aneurysm expansion by increasing SMC proliferation [31].

Recent studies have proposed multiple miRNAs as useful biomarkers for cardiovascular diseases [32,33,34]. The hemodynamic alterations and genetic background of patients also need to be taken into consideration before drawing conclusions on the role of particular miRNAs in aneurysm, as these factors can attribute to the heterogeneity in patients. This is confirmed in our study, as we did observe variation in expression profiles between our study and a recent study done using ascending aorta patients with stenotic tricuspid (TAVA) or bicuspid aortic valve (BAV) tissues [35]. Also, it is of further significance to note that there exists a variability in the expression of miRNAs between site of aneurysmal tissue (i.e., aortic tissue body (site of maximum dilation) and neck (non-dilated aorta)) [36]. From our observation in the current study, we deduce that individual pathologies should be uniquely considered with respect to miRNA expression pattern, and any therapeutic intervention by miRNAs should be designed specifically for each disease. 

One of the potential treatments for TAA might be the use of miRNA mimics or miRNA antagomirs, specifically for miR-126-3p, miR-204, and miR-486-5p, as expression of these miRNAs were found to be upregulated in TAA specimens. Out of these three miRNAs, miR-126 seems to have the most prominent role in vascular integrity. The deletion of miR-126 in mice led to the formation of leaky blood vessels and caused defects in angiogenesis. However, the function of miR-126 in TAA and its use as a potential biomarker of TAA in vivo has not yet been studied. Importantly, it was reported that atorvastatin can selectively affect miR-126-3p expression and aspirin plays a role in downregulation of the miR-126-3p [37,38,39]. However, these modulations in the miR expression can vary based on the cell type and function. On the other hand, miRNAs like miR-145, miR-146a, and miR-486-5p were differentially expressed in AAA, and miR-146 had a more specific role for AAA. It was suggested that angiotensin receptor blockers and statins can downregulate miR-146a expression level via Interleukin-1 receptor-associated kinase 1 (IRAK-1), TNF Receptor Associated Factor 6 (TRAF6), and Toll-like receptor -4 (TLR-4) pathways [40]. In addition, hormonal therapy also could be an option for manipulation of miR-146a expression level, as estrogen moderates miR’s expression and progesterone leads to a delay in miR-146a expression [41,42]. Overall, it seems that treatment with statin group medication is a reasonable option for suppression of both miR-126-3p and miR-146a expression levels, leading to prevention of aortic diseases. However, manipulation of miRs expression by medication is complex because they have multiple and controversial interaction in the human body [43].

**Figure 6:**
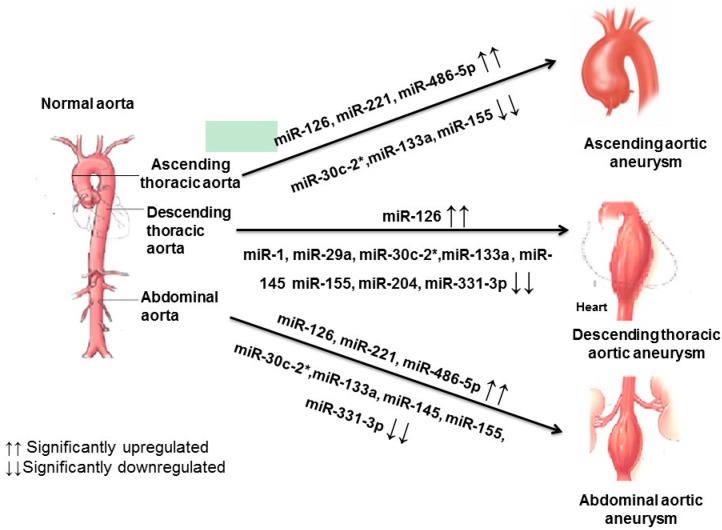
Dominant cardiovascular miRNAs patterns observed in TAA and AAA.

In conclusion, we identified unique miRNA signatures for TAA and AAA and clarified profiles in expression that represent common features in both pathologies (Figure 6). Importantly, this work validates prior reports in the literature and provides further justification for the pursuit of employing miRNA detection as a novel diagnostic/prognostic approach of aneurysmal disease and the development of methods to regulate their expression as therapeutic targets.

## 4. Materials and Methods

### 4.1. Patient Demographics

The study population consists of three patient groups: TAA, AAA, and controls. Aortic tissue specimens were obtained from TAA (*n* = 7) and AAA (*n* = 3) patients at the time of elective aortic surgery (Table 1). These fresh tissues were collected from the Biorepository; Clinical and Translational Science Institute (CTSI) (IRB 201300201); and Department of Surgery, University of Florida (IRB 614-2008). Normal control artery (aortic diameters <50–55 mm, i.e., below the threshold of intervention) consisting of non-aneurysmal ascending aortic specimens <45 mm (banked tissue) (*n* = 8) (IRB PR007020120) were obtained from the Department of Cardiothoracic Surgery, University of Pittsburgh (Table 1). All procedures performed in studies involving human participants were in accordance with the ethical standards of the institutional and/or national research committee and with the 1975 Helsinki declaration and its later amendments in 2000 (5) or comparable ethical standards. Informed consent was obtained from all patients for being included in the study.

### 4.2. Sample Preparation and miRNA Analysis

Resected aortic tissue specimens were homogenized in cell disruption buffer using a Qiagen TissueRuptor kit. Total RNA was isolated by following mirVana miRNA isolation kit protocol (Applied Biosystems, Foster City, CA, USA). The PCR conditions for reverse transcription were 16 °C for 30 min, 42 °C for 30 min, 85 °C for 5 min, and held at 4 °C. For quantitative PCR, assays were conducted in duplicate for each sample. The real-time PCR assay was performed using the 7500 Fast Real-Time PCR System (Applied Biosystems) for miRNAs (miR-1, miR-21, miR-29a, miR-30c-2*, miR-124a, miR-126, miR-133a miR-145, miR-146a, miR-155, miR-204, miR-221, miR-222 miR-331-3p, and miR-486-5p); and RNU44, internal control to analyze specific miRNA expression following the 2^−ΔΔ*C*t^ method. miRNAs were analyzed with TaqMan MicroRNA Assays (Applied Biosystems, Foster City, CA, USA). The two-step procedure was as follows: initial denaturation for 10 min at 95 °C, followed by 40 cycles of 15 s at 95 °C, and 60 s at 60 °C [44].

### 4.3. Microarray and Bioinformatics Analysis

The Human miRNA Microarray (miRBase database Release 19.0, 8 × 60 K) was selected for the assessment of 2006 human miRNAs on the healthy control and TAA transcriptome. One hundred nanograms of total RNA from TAA samples (*n* = 5) and healthy control samples (*n* = 3) were labeled and hybridized using the miRNA Complete Labeling and Hyb Kit (Agilent Technologies, Santaclara, CA, USA) according to the manufacturer’s instructions. In short, RNA samples were first dephosphorylated using calf intestinal phosphatase and denatured using dimethylsulfoxide. Next, RNA samples were labeled with cyanine 3-pCp using T4 RNA ligase for 2 h at 16 °C and hybridized onto the miRNA microarrays for 20 h at 55 °C in a hybridization oven at 20 rpm. The slides were scanned and Agilent Feature Extraction Software was used to generate text files for each RNA hybridization chip. All analyses were performed using R3.0.0 software [45]. Background adjustment, summarization, and quantile normalization were performed using Limma package. Normalization was made using the Robust Multichip Average pre-normalization algorithm [46]. Data quality was assessed using various Quality control (QC) charts (Density & Intensity plot). Using biological replicate microarrays, each probe-set signal value from TAA samples (*n* = 5) was compared to the probe-set signal value of healthy control samples (*n* = 3) to generate gene expression ratios.

### 4.4. Statistical Analysis

Prism for Windows, Version 5.0 (GraphPad Software Inc., La Jolla, CA, USA) was used for analysis of qRT-PCR data, and *p* < 0.05 was considered significant. Genes were considered to be differentially expressed at *p* < 0.05 and fold change >1.5. An unpaired, nonparametric two-tailed Student’s *t-*test was used to compare two independent groups (TAA vs. control, AAA vs. control, and TAA vs. AAA) for miRNA expression analyses.

### 4.5. Study Limitations

This is an observational study and has some limitations. (a) A small number of samples were available for this study and this study may not reflect the complete gender-based analysis of TAA and AAA; for such evaluations, future research is needed with large numbers of patient-based studies. (b) Due to the difficulties in obtaining the normal control and samples from patients, this study incorporated groups of patients with unequal number. (c) Being an observational study, the study is limited by its inability to perform validations. In order to validate miRNAs in relation to growth of each aneurysms we need to include a large number of patients in each group, and inclusion of matched controls would be needed, which were beyond the scope of this study.

## Figures and Tables

**Table 1:** Summary of clinical details of human tissue samples used for quantitative reverse transcription polymerase chain reaction (qRT-PCR) and microarray analysis.

	Body Site	Ascending/Descending	Surgical Diagnosis/Tissue Location			Hypertension/Smoking
	**Abdominal**					
1	AAA		AAA, for repair, aneurysm tissue, vessel wall			Y/Y
2	AAA		juxtarenal ascending aortic aneurysm, aortic wall			Y/Y
3	AAA		AAA, for repair			Y/Y
	**Thoracic**					
1	TAA	ascending	aortic arch repair, aorta, aneurysm wall			UK/N
2	TAA	ascending	ascending arch aneurysm, aorta			Y/Y
3	TAA	ascending	ascending/arch aneurysm, aorta			Y/Y
4	TAA	ascending	ascending aneurysm repair, aorta			N/Y
5	TAA	descending	thoracic aneurysm repair, aorta			N/N
6	TAA	ascending	ascending TAA, aorta			Y/N
7	TAA	descending	aortic aneurysm above a previous EVAR, aorta			Y/Y
8	DTAA	descending	descending TAA			
9	TAA	ascending	ascending TAA			
10	TAA	ascending	ascending TAA			
11	TAA	ascending	TAA			
	**Aorta**		**Operation**	**Aortic Diameter**	**Diagnosis**	
1	Control		Heart transplant recipient	<32 mm	Ischemic cardio myopathy	
2	Control		Heart transplant recipient	<32 mm	Uhl’s anomaly	
3	Control		Ascending aorta, total arch, descending aorta replacement	34 mm	Kommerell’s diverticulum aneurysm	
4	Control		Heart transplant recipient	<32 mm	Ischemic cardiomyopathy	
5	Control		Redo root/asc/hemi arch replacement	42 mm	Bicuspid aortic valve	
6	Control		Heart transplant recipient	<32 mm	Ischemic cardiomyopathy	
7	Control		Aortic valve replacement/root and ascending aorta replacement	40 mm	Severe aortic insufficiency	
8	Control		Heart transplant recipient	<32 mm	Congenital tricuspid atresia	

All 22 tissues (3 abdominal, 11 thoracic, 8 controls) are used for qRT-PCR analysis and 5 TAA tissues (2, 3, 4, 5, 8) are used for microarray analysis. AAA, abdominal aortic aneurysm; TAA, thoracic aortic aneurysm; DTAA, descending TAA; Asc, ascending; EVAR, endovascular aneurysm repair; Y, yes; N, No; and UK, unknown.

**Table 2:** Summary of differential expression patterns of microRNA (miRNA) in thoracic aortic aneurysm (TAA) and abdominal aortic aneurysm (AAA) by qRT-PCR.

	miRNA	TAA	AAA
1	miR-1	Downregulated	No alteration
2	miR-21	No alteration	No alteration
3	miR-29a	Downregulated	No alteration
4	miR-30c-2*	Downregulated	Downregulated
5	miR-124	No alteration	No alteration
6	**miR-126**	Upregulated	Upregulated
7	miR-133a	Downregulated	No alteration
8	miR-145	No alteration	Downregulated
9	miR-146	No alteration	Upregulated
10	**miR-155**	Downregulated	Downregulated
11	**miR-204**	Downregulated	Downregulated
12	miR-221	Upregulated	No alteration
13	miR-222	No alteration	No alteration
14	miR-331–3p	No alteration	Downregulated
15	**miR-486–5p**	Upregulated	Upregulated

Instances where miR-126 and miR-486–5p were upregulated in both TAA and AAA are indicated in bold font. Similarly, instances where miR-30c-2*, miR-155, and miR-204 were downregulated in both TAA and AAA are indicated in bold font.

**Table 3:** Upregulated TAA miRNAs in Agilent’s Human miRNA microarray Release 19.0 *.

Serial Number	miRNA	Fold Change	*p* Value
1	hsa-miR-15b-5p	1.66	0.021
2	**hsa-miR-16-5p**	**1.93**	**0.0072**
3	hsa-miR-21-3p	1.58	0.036
4	**hsa-miR-21-5p**	**3.67**	**0.0079**
5	**hsa-miR-25-3p**	**2.12**	**0.0079**
6	**hsa-miR-106b-5p**	**1.54**	**0.00071**
7	**hsa-miR-126-3p**	**3.88**	**0.0091**
8	hsa-miR-146b-5p	2.72	0.013
9	**hsa-miR-185-5p**	**1.88**	**0.0032**
10	**hsa-miR-223-3p**	**5.81**	**0.0081**
11	**hsa-miR-451a**	**17.91**	**0.00016**
12	hsa-miR-486-5p	4.00	0.018
13	hsa-miR-494	1.72	0.021
14	hsa-miR-642a-3p	2.32	0.032
15	**hsa-miR-1260b**	**2.32**	**0.0019**
16	**hsa-miR-1260a**	**3.62**	**0.00004**
17	**hsa-miR-1268a**	**1.60**	**0.0051**
18	hsa-miR-4284	1.63	0.048
19	**hsa-miR-4286**	**1.91**	**0.00050**
20	hsa-miR-4306	1.73	0.017
21	hsa-miR-4454	1.83	0.014
22	hsa-miR-4459	1.69	0.42
23	hsa-miR-4763-3p	1.96	0.044
24	hsa-miR-6090	1.79	0.043

* miRNAs expression changes in TAA with *p* value < 0.01 are indicated in bold font. Out of 24 miRNAs, 12 miRNAs (50%) were significantly upregulated in TAA specimens by microarray.

**Table 4:** Downregulated TAA miRNAs in Agilent’s Human miRNA microarray Release 19.0 ^†^.

Serial Number	miRNA	Fold Change	*p* Value
1	hsa-miR-125b-5p	0.41	0.041
2	hsa-miR-204-5p	0.47	0.035
**3**	**hsa-miR-371b-5p**	**0.50**	**0.00078**
**4**	**hsa-miR-572**	**0.50**	**0.0020**
**5**	**hsa-miR-638**	**0.52**	**0.0035**
**6**	**hsa-miR-1227-5p**	**0.53**	**0.0025**
**7**	**hsa-miR-1273g-3p**	**0.54**	**0.00018**
**8**	**hsa-miR-1273f**	**0.54**	**0.00002**
**9**	**hsa-miR-2861**	**0.56**	**0.0085**
**10**	**hsa-miR-3135b**	**0.56**	**0.0014**
**11**	**hsa-miR-3652**	**0.59**	**0.00074**
12	hsa-miR-3665	0.61	0.014
**13**	**hsa-miR-3960**	**0.61**	**0.0019**
14	hsa-miR-4324	0.62	0.039
**15**	**hsa-miR-4507**	**0.62**	**0.0099**
**16**	**hsa-miR-4787-5p**	**0.63**	**0.0051**
**17**	**hsa-miR-5001-5p**	**0.64**	**0.00065**
**18**	**hsa-miR-6068**	**0.64**	**0.0022**
**19**	**hsa-miR-6089**	**0.65**	**0.00013**
**20**	**hsa-miR-6125**	**0.66**	**0.00096**
**21**	**hsa-miR-6724-5p**	**0.66**	**0.0029**

^†^ miRNAs expression changes in TAA with *p* value < 0.01 are indicated in bold font. Out of 21 miRNAs, 17 miRNAs were significantly downregulated in TAA specimens by microarray.

## References

[1] Meszaros I., Morocz J., Szlavi J., Schmidt J., Tornoci L., Nagy L., Szep L. (2000). Epidemiology and clinicopathology of aortic dissection. Chest.

[2] Bozeman M.C., Ross C.B. (2012). Intra-abdominal hypertension and abdominal compartment syndrome in association with ruptured abdominal aortic aneurysm in the endovascular era: Vigilance remains critical. Crit. Care Res. Pract..

[3] Ye Z., Bailey K.R., Austin E., Kullo I.J. (2016). Family history of atherosclerotic vascular disease is associated with the presence of abdominal aortic aneurysm. Vasc. Med..

[4] Peshkova I.O., Schaefer G., Koltsova E.K. (2016). Atherosclerosis and aortic aneurysm: Is inflammation a common denominator?. FEBS J..

[5] Abdulkareem N., Smelt J., Jahangiri M. (2013). Bicuspid aortic valve aortopathy: Genetics, pathophysiology and medical therapy. Interact. Cardiovasc. Thorac. Surg..

[6] Cury M., Zeidan F., Lobato A.C. (2013). Aortic disease in the young: Genetic aneurysm syndromes, connective tissue disorders, and familial aortic aneurysms and dissections. Int. J. Vasc. Med..

[7] Elefteriades J.A. (2002). Natural history of thoracic aortic aneurysms: Indications for surgery, and surgical versus nonsurgical risks. Ann. Thorac. Surg..

[8] Seeger T., Boon R.A. (2016). MicroRNAs in cardiovascular ageing. J. Physiol..

[9] Chen J.F., Murchison E.P., Tang R., Callis T.E., Tatsuguchi M., Deng Z., Rojas M., Hammond S.M., Schneider M.D., Selzman C.H. (2008). Targeted deletion of Dicer in the heart leads to dilated cardiomyopathy and heart failure. Proc. Natl. Acad. Sci. USA.

[10] Saxena A., Tabin C.J. (2010). Mirna-processing enzyme dicer is necessary for cardiac outflow tract alignment and chamber septation. Proc. Natl. Acad. Sci. USA.

[11] Zhao Y., Samal E., Srivastava D. (2005). Serum response factor regulates a muscle-specific microRNA that targets Hand2 during cardiogenesis. Nature.

[12] Cordes K.R., Sheehy N.T., White M.P., Berry E.C., Morton S.U., Muth A.N., Lee T.H., Miano J.M., Ivey K.N., Srivastava D. (2009). miR-145 and miR-143 regulate smooth muscle cell fate and plasticity. Nature.

[13] Albinsson S., Suarez Y., Skoura A., Offermanns S., Miano J.M., Sessa W.C. (2010). MicroRNAs are necessary for vascular smooth muscle growth, differentiation, and function. Arterioscler. Thromb. Vasc. Biol..

[14] Small E.M., Olson E.N. (2011). Pervasive roles of microRNAs in cardiovascular biology. Nature.

[15] Boon R.A., Seeger T., Heydt S., Fischer A., Hergenreider E., Horrevoets A.J., Vinciguerra M., Rosenthal N., Sciacca S., Pilato M. (2011). MicroRNA-29 in aortic dilation: Implications for aneurysm formation. Circ. Res..

[16] Kin K., Miyagawa S., Fukushima S., Shirakawa Y., Torikai K., Shimamura K., Daimon T., Kawahara Y., Kuratani T., Sawa Y. (2012). Tissue- and plasma-specific microRNA signatures for atherosclerotic abdominal aortic aneurysm. J. Am. Heart Assoc..

[17] Zhang P., Huang A., Ferruzzi J., Mecham R.P., Starcher B.C., Tellides G., Humphrey J.D., Giordano F.J., Niklason L.E., Sessa W.C. (2012). Inhibition of microRNA-29 enhances elastin levels in cells haploinsufficient for elastin and in bioengineered vessels—Brief report. Arterioscler. Thromb. Vasc. Biol..

[18] Jones J.A., Stroud R.E., O’Quinn E.C., Black L.E., Barth J.L., Elefteriades J.A., Bavaria J.E., Gorman J.H., Gorman R.C., Spinale F.G. (2011). Selective microRNA suppression in human thoracic aneurysms relationship of miR-29a to aortic size and proteolytic induction. Circ. Cardiovasc. Genet..

[19] Liao M., Zou S., Weng J., Hou L., Yang L., Zhao Z., Bao J., Jing Z. (2011). A microRNA profile comparison between thoracic aortic dissection and normal thoracic aorta indicates the potential role of microRNAs in contributing to thoracic aortic dissection pathogenesis. J. Vasc. Surg..

[20] Pahl M.C., Derr K., Gabel G., Hinterseher I., Elmore J.R., Schworer C.M., Peeler T.C., Franklin D.P., Gray J.L., Carey D.J. (2012). MicroRNA expression signature in human abdominal aortic aneurysms. BMC Med. Genom..

[21] Liu X., Cheng Y., Zhang S., Lin Y., Yang J., Zhang C. (2009). A necessary role of miR-221 and miR-222 in vascular smooth muscle cell proliferation and neointimal hyperplasia. Circ. Res..

[22] Merk D.R., Chin J.T., Dake B.A., Maegdefessel L., Miller M.O., Kimura N., Tsao P.S., Iosef C., Berry G.J., Mohr F.W. (2012). miR-29b participates in early aneurysm development in marfan syndrome. Circ. Res..

[23] Wang S., Aurora A.B., Johnson B.A., Qi X., McAnally J., Hill J.A., Richardson J.A., Bassel-Duby R., Olson E.N. (2008). The endothelial-specific microRNA miR-126 governs vascular integrity and angiogenesis. Dev. Cell.

[24] Wu X.Y., Fan W.D., Fang R., Wu G.F. (2014). Regulation of microRNA-155 in endothelial inflammation by targeting nuclear factor (NF)-κB p65. J. Cell. Biochem..

[25] Duisters R.F., Tijsen A.J., Schroen B., Leenders J.J., Lentink V., van der Made I., Herias V., van Leeuwen R.E., Schellings M.W., Barenbrug P. (2009). MiR-133 and miR-30 regulate connective tissue growth factor implications for a role of microRNAs in myocardial matrix remodeling. Circ. Res..

[26] Taganov K.D., Boldin M.P., Chang K.J., Baltimore D. (2006). NF-κB-dependent induction of microRNA miR-146, an inhibitor targeted to signaling proteins of innate immune responses. Proc. Natl. Acad. Sci. USA.

[27] Boettger T., Beetz N., Kostin S., Schneider J., Kruger M., Hein L., Braun T. (2009). Acquisition of the contractile phenotype by murine arterial smooth muscle cells depends on the miR143/145 gene cluster. J. Clin. Investig..

[28] Elia L., Quintavalle M., Zhang J., Contu R., Cossu L., Latronico M.V., Peterson K.L., Indolfi C., Catalucci D., Chen J. (2009). The knockout of miR-143 and -145 alters smooth muscle cell maintenance and vascular homeostasis in mice: Correlates with human disease. Cell Death Differ..

[29] Xin M., Small E.M., Sutherland L.B., Qi X., McAnally J., Plato C.F., Richardson J.A., Bassel-Duby R., Olson E.N. (2009). MicroRNAs miR-143 and miR-145 modulate cytoskeletal dynamics and responsiveness of smooth muscle cells to injury. Genes Dev..

[30] Guo X., Guo L., Ji J., Zhang J., Chen X., Cai Q., Li J., Gu Q., Liu B., Zhu Z. (2010). MiRNA-331-3p directly targets E2F1 and induces growth arrest in human gastric cancer. Biochem. Biophys. Res. Commun..

[31] Maegdefessel L., Azuma J., Toh R., Deng A., Merk D.R., Raiesdana A., Leeper N.J., Raaz U., Schoelmerich A.M., McConnell M.V. (2012). MicroRNA-21 blocks abdominal aortic aneurysm development and nicotine-augmented expansion. Sci. Transl. Med..

[32] Wang J., Chen J., Sen S. (2016). MicroRNA as biomarkers and diagnostics. J. Cell. Physiol..

[33] Wang G.K., Zhu J.Q., Zhang J.T., Li Q., Li Y., He J., Qin Y.W., Jing Q. (2010). Circulating microRNA: A novel potential biomarker for early diagnosis of acute myocardial infarction in humans. Eur. Heart J..

[34] Ai J., Zhang R., Li Y., Pu J., Lu Y., Jiao J., Li K., Yu B., Li Z., Wang R. (2010). Circulating microRNA-1 as a potential novel biomarker for acute myocardial infarction. Biochem. Biophys. Res. Commun..

[35] Albinsson S., Della Corte A., Alajbegovic A., Krawczyk K.K., Bancone C., Galderisi U., Cipollaro M., de Feo M., Forte A. (2017). Patients with bicuspid and tricuspid aortic valve exhibit distinct regional microRNA signatures in mildly dilated ascending aorta. Heart Vessels.

[36] Biros E., Moran C.S., Rush C.M., Gabel G., Schreurs C., Lindeman J.H., Walker P.J., Nataatmadja M., West M., Holdt L.M. (2014). Differential gene expression in the proximal neck of human abdominal aortic aneurysm. Atherosclerosis.

[37] Zhang Q., Kandic I., Kutryk M.J. (2011). Dysregulation of angiogenesis-related microRNAs in endothelial progenitor cells from patients with coronary artery disease. Biochem. Biophys. Res. Commun..

[38] Zhang Y., Yang P., Sun T., Li D., Xu X., Rui Y., Li C., Chong M., Ibrahim T., Mercatali L. (2013). miR-126 and miR-126* repress recruitment of mesenchymal stem cells and inflammatory monocytes to inhibit breast cancer metastasis. Nat. Cell. Biol..

[39] Shi H., Chen L., Wang H., Zhu S., Dong C., Webster K.A., Wei J. (2013). Synergistic induction of miR-126 by hypoxia and hdac inhibitors in cardiac myocytes. Biochem. Biophys. Res. Commun..

[40] Takahashi Y., Satoh M., Minami Y., Tabuchi T., Itoh T., Nakamura M. (2010). Expression of miR-146a/b is associated with the toll-like receptor 4 signal in coronary artery disease: Effect of renin-angiotensin system blockade and statins on miRNA-146a/b and toll-like receptor 4 levels. Clin. Sci..

[41] Dai R., Phillips R.A., Zhang Y., Khan D., Crasta O., Ahmed S.A. (2008). Suppression of LPS-induced interferon-γ and nitric oxide in splenic lymphocytes by select estrogen-regulated microRNAs: A novel mechanism of immune modulation. Blood.

[42] Sun Y., Cai J., Ma F., Lu P., Huang H., Zhou J. (2012). miR-155 mediates suppressive effect of progesterone on TLR3, TLR4-triggered immune response. Immunol. Lett..

[43] Aalaei-Andabili S.H., Fabbri M., Rezaei N. (2013). Reciprocal effects of toll-like receptors and miRNAs on biological processes in human health and disease: A systematic review. Immunotherapy.

[44] Nahid M.A., Rivera M., Lucas A., Chan E.K., Kesavalu L. (2011). Polymicrobial infection with periodontal pathogens specifically enhances microRNA miR-146a in ApoE^−/−^ mice during experimental periodontal disease. Infect. Immun..

[45] TEAM RC R: A Language and Environment for Statistical Computing. http://www.R-project.org.

[46] Irizarry R.A., Hobbs B., Collin F., Beazer-Barclay Y.D., Antonellis K.J., Scherf U., Speed T.P. (2003). Exploration, normalization, and summaries of high density oligonucleotide array probe level data. Biostatistics.

